# Direct reduction of high-grade lumbosacral spondylolisthesis with anterior cantilever technique - surgical technique note and preliminary results

**DOI:** 10.1186/s12891-021-04439-7

**Published:** 2021-06-18

**Authors:** Kao-Chang Tu, Cheng-Min Shih, Kun-Hui Chen, Chien-Chou Pan, Fuu-Cheng Jiang, Cheng-En Hsu, Yun-Ming Wang, Cheng-Hung Lee

**Affiliations:** 1 Department of Orthopaedic Surgery, Taichung Veterans General Hospital, 1650 Taiwan Boulevard Sect. 4, Taichung, 40705 Taiwan; 2 PhD Degree Program of Biomedical Science and Engineering, College of Biological Science and Technology, National Yang Ming Chiao Tung University, Hsinchu, 300 Taiwan; 3 Department of Physical Therapy, Hungkuang University, Taichung, Taiwan; 4 Department of Nursing, Jenteh Junior College of Medicine, Nursing and Management, Miaoli, Taiwan; 5 Department of Computer Science & Information Engineering, College of Computing and Informatics, Providence University, Taichung, Taiwan; 6 Department of Rehabilitation Science, Jenteh Junior College of Medicine, Nursing and Management, Miaoli, Taiwan; 7 Department of Computer Science, Tunghai University, Taichung, Taiwan; 8 Sports Recreation and Health Management Continuing Studies-Bachelor’s Degree Completion Program, Tunghai University, Taichung, Taiwan; 9 Institute of Molecular Medicine and Bioengineering, College of Biological Science and Technology, National Yang Ming Chiao Tung University, Hsinchu, Taiwan; 10 Department of Food Science and Technology, Hungkuang University, Taichung, Taiwan

**Keywords:** Cantilever, High grade spondylolisthesis, Mono-segment instrumentation, Reduction, Sagittal balance, Severe spondylolisthesis

## Abstract

**Backgrounds:**

Surgical reduction for high-grade spondylolisthesis is beneficial for restoring sagittal balance and improving the biomechanical environment for arthrodesis. Compared to posterior total laminectomy and long instrumentation, anterior lumbar inter-body fusion (ALIF) is less invasive and has the biomechanical advantage of restoring the original disk height and increasing lumbar lordosis, thus improving sagittal balance. However, the application of ALIF is still limited in treating low-grade spondylolisthesis. In this study, we developed a new technique termed anterior cantilever procedure to directly reduce the slippage of high-grade lumbosacral spondylolisthesis. The purpose of our study was to investigate the surgical outcomes of the anterior cantilever procedure followed by ALIF and posterior mono-segment instrumented fixation in high-grade spondylolisthesis.

**Methods:**

All patients with high-grade spondylolisthesis who underwent anterior cantilever procedure followed by anterior lumbar inter-body fusion (ALIF) and posterior mono-segment instrumented fixation between November 2006 and July 2017 were enrolled in our study. The slip percentage, Dubousset’s lumbosacral angle, pelvic tilt, sacral slope, pelvic incidence, and sagittal alignment were measured pre-operatively and postoperatively at the last follow-up. Surgery time, blood loss, complications, and hospital stay were also collected and analysed.

**Results:**

A total of 11 consecutive patients with high-grade spondylolisthesis patients were included and analysed. All of the high-grade spondylolisthesis in our series occurred at the L5-S1 level. The median age was 37 years, and the median follow-up duration was 36 months. The average slip reduction was 30% (60 to 30%, *P* < 0.01), and the average correction of Dubousset’s lumbosacral angle was 13.8° (84.1° to 97.9°, *P* < 0.01). The median intra-operative blood loss was 300 mL. All patients attained improved sagittal balance after the operation and achieved solid fusion within 9 months after surgery. No incidences of implant failure, permanent neurological deficit, or pseudarthrosis were recorded at the last follow-up.

**Conclusions:**

Anterior cantilever procedure followed by ALIF and posterior mono-segment instrumented fixation is a valid procedure for treating high-grade spondylolisthesis. It achieved a high fusion rate, partially reduced slippage, and significantly improved lumbosacral angle, while minimizing common complications, such as pseudarthrosis, nerve traction injury, excessive soft tissue dissection, and blood loss in posterior reduction procedures. However, posterior instrumentation is still required to the structural stability in the ALIF procedure.

**Level of evidence:**

IV

## Backgrounds

High-grade spondylolisthesis is defined as slips greater than 50% and Meyerding grade III or higher. It is a rare condition accounting for approximately 1% of all spinal spondylolisthesis cases [1]. Theoretically, surgical reduction of spondylolisthesis restores sagittal balance, improves the biomechanical environment for arthrodesis, maintains stable standing position with a minimum expenditure, and decreases the compensatory mechanism of the hip and knee [2–4]. However, the role of surgical reduction in high-grade spondylolisthesis is still controversial because of its potential complications, including neurologic deficits, prolonged operative time, and implant failure [5–8]. In a recent study which evaluated the effect of surgical reduction from 60 high-grade spondylolisthesis patients, surgical reduction in high- to low-grade slip was found to be more effective for maintaining and restoring a normal pelvic balance postoperatively [9].

In comparison to posterior total laminectomy and long instrumentation, anterior lumbar inter-body fusion (ALIF) has the following advantages in the treatment of high-grade spondylolisthesis patients: low possibility of neural injury during the operation; biomechanical simplicity to widen the vertebral bodies to their original disk height, to restore the lumbar lordosis as well as the sagittal balance; comparatively less blood loss; short hospital stay; low complication rate; and a high fusion rate. In order to attain the abovementioned advantages, some authors advocated ALIF and percutaneous pedicle screws to treat isthmic spondylolisthesis [10–12]. Though good to excellent results have been reported, to date, the application of ALIF is still limited in the treatment of low-grade lumbar spondylolisthesis [11–13].

In this study, we developed a reduction technique termed “anterior cantilever procedure” to reduce high-grade spondylolisthesis via the anterior approach. The anterior cantilever procedure followed by ALIF and posterior mono-segment instrumented fixation may correct the deformity and reduce the nerve injury risk during the operation in high-grade spondylolisthesis. The purpose of our study was to introduce the surgical technique and investigate the surgical outcomes of this approach.

## Methods

### Patient enrollment

The medical and radiographic records of patients with symptomatic high-grade lumbosacral spondylolisthesis who underwent anterior cantilever procedure followed by ALIF and posterior mono-segment instrumented fixation between November 2006 and July 2017 were collected retrospectively.

The inclusion criteria were patients with normal mental health and a complete set of data from the functional status questionnaires and measurements used in this study. The exclusion criteria were pathologic fractures, previous lumbar spine surgery, or diagnosis of degenerative scoliosis.

### Data collection

All patients had preoperative radiographs, spinal computed tomography (CT) scans, as well as magnetic resonance imaging (MRI) of the lumbar spine. Dynamic lumbar spine radiography was done to confirm the diagnosis. The determination of fusion success was independently assessed by a blinded radiologist according to the following criteria: the absence of motion between the fusion segments on lateral flexion-extension views, no radiolucency in the disc space and formation of a bone bridge connecting the vertebral bodies above and below.

Radiographic parameters included slip percentage, Dubousset’s lumbosacral angle (LSA) [14], sacral slope (SS), pelvic incidence (PI), pelvic tilt (PT) [2], lumbar lordosis (LL), and sagittal vertical axis (SVA) [15]. All parameters were on the picture archiving and communication system.

Functional status questionnaires including the Oswestry Disability Index (ODI), visual analogue scale (VAS), and European Quality of Life questionnaire (EQ-5D) were used to assess the preoperative and postoperative functional status. All data were collected by a blinded observer who had 3 years’ experience of spinal surgery.

#### Surgical technique

All patients completed the two-stage operation under general anaesthesia on the same day. The first stage was ALIF with anterior cantilever procedure; the second stage was posterior mono-segment instrumented fixation.

**Fig. 1:**
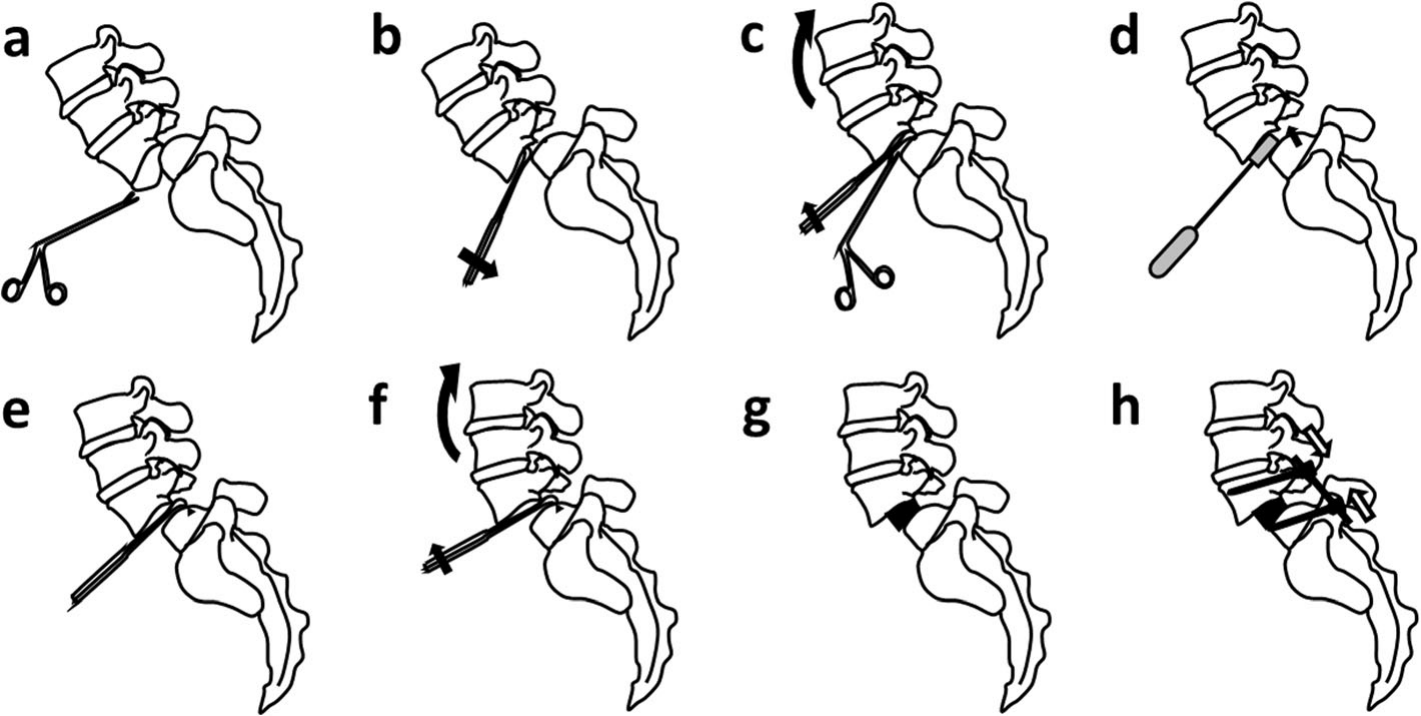
Graphic illustration of the operative process. **A** The L5-S1 level was identified, and the anterior longitudinal ligament, annulus fibrosis, and partial disc were removed. **B** First cantilever: open anterior disc space. **C** Complete discectomy and endplate preparation. **D** The disc space was dilated, the foramen height was restored, and the posterior facet joints were open or mobilised by the trial cage. **E** The Cobb elevator blade was placed into the posterior surface of the sacral dome as a hinge. **F** Second cantilever: the posterior elements were released, and spondylolisthesis was reduced. **G** Anterior lumbar inter-body fusion. **H** Supplemented with posterior instrumentation using the Wiltse approach

In the first stage of the operation, the patient was positioned supine on the radiolucent operating Table. A lateral radiograph by C-arm was obtained for marking the L5-S1 level; the abdomen was then prepared and draped following a standard sterile procedure. A straight midline incision of approximately 8 cm was made in the lower abdomen and the abdominal muscles were gently spread apart; the retroperitoneum was approached with blunt dissection and the peritoneal sac was retracted to the right. The psoas muscle and iliac vessels were then visualised and carefully retracted laterally. After the L5-S1 level was identified, specific retractors for anterior approach were used to maintain the exposure. The median sacral vessels were coagulated with bipolar coagulation forceps and the anterior cantilever procedure was performed after removal of the anterior longitudinal ligament, thickened fibrotic anterior annulus, and anterior disc (Fig. 1A). The anterior cantilever procedure for spondylolisthesis reduction was initiated by inserting a Cobb elevator blade to open and clear the middle and posterior parts of the disc (Fig. 1B). After completing meticulous endplate preparation (Fig. 1C), a trial cage was inserted temporarily to dilate the disc space and the L5-S1 facet joints were then removed (Fig. 1D). A Cobb elevator blade was then placed at the posterior margin of the sacral dome as a hinge (Fig. 1E). The Cobb elevator was elevated superiorly working as a cantilever to further release the tension of the posterior annulus and reduce the slippage (Fig. 1F).

**Fig. 2:**
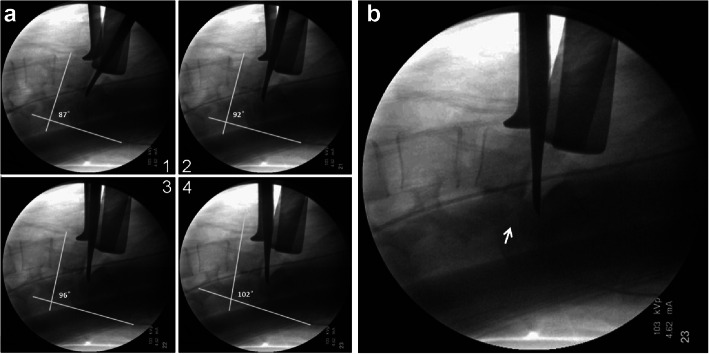
Images of the representative case. **A** Pre-operative plain films revealed Dubousset’s lumbosacral angle (LSA) of 66° and slip percentage of 75%. **B** Magnetic resonance image showing a thickened anterior portion of the L5-S1 annulus and reduced disc signal intensity. **C** At 9 months postoperatively, plain films of case 1 showed correction of the L5-S1 spondylolisthesis and solid inter-body fusion (black arrow)

The resistance of L5, S1 and the posterior complex was gradually released after performing repetitive anterior cantilever procedure 3–5 times (Fig. 2). Care should be taken during placement of the Cobb elevator blade at the posterior margin of the sacral dome to prevent intrusion into the dura.

After appropriate release of the L5-S1 space with the anterior cantilever procedure, a large, wedge-shaped lordotic design cage with autogenous bone marrow aspirate and allogenous cancellous bone graft as the bone supplement in cage, can be symmetrically placed at the desired level (Fig. 1G). Finally, the position of the inter-body cage and reduction of the L5-S1 spondylolisthesis were confirmed via C-arm fluoroscopy. Two 3.5-mm cancellous screws were inserted at the anterior corner of the S1 endplate to prevent cage dislocation during repositioning for posterior instrumentation. After checking for bleeders and removal of retractors, the wound was closed layer by layer.

**Fig. 3:**
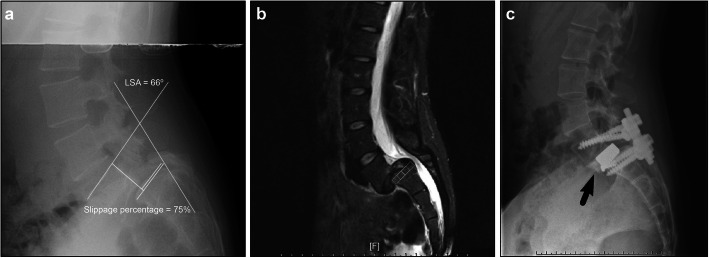
Repetitve anterior cantilever procedure restored the lumbar lordosis and the foraminal height. **A** Dubousset’s LSA increased gradually during the anterior cantilever procedure (steps 1–4). **B** The *pars interarticularis* was obviously distracted (white arrow) with increased foraminal height

In the second stage of operation, the patient was placed in the prone position, a posterior midline skin incision was made, and Wiltse muscle-sparing approach was adopted. The pedicle screws were bilaterally inserted into the L5 and S1 pedicles and two lordotic rods were placed in a suitable position. The pedicle screws and rods were carefully compressed to create lumbar lordosis (Fig. 1H). The screw position and lumbar spine alignment were confirmed via C-arm fluoroscopy; the posterior approach was then closed. In patients with severe central stenosis, laminectomy was performed to relieve the symptoms. Postoperative plain radiographs were obtained at regular intervals to assess inter-body fusion and slip correction (Fig. 3).

### Postoperative evaluation

Patients were followed up at 2 weeks, 1 month, 3 months, 6 months, 1 year, and 2 years after operation. Knight-spinal lumbosacral orthosis application was used for 8 weeks postoperatively.

Postoperative Dubousset’s lumbosacral angle (LSA) [14], sacral slope (SS), pelvic incidence (PI), pelvic tilt (PT) [2], lumbar lordosis (LL) and sagittal vertical axis (SVA) [15], Functional status questionnaires including the Oswestry Disability Index (ODI), visual analogue scale (VAS), and European Quality of Life questionnaire (EQ-5D) were measured at the last postoperative follow-up visit. The clinical outcome was graded according to the modified Macnab criteria.

### Statistical analysis

Data analysis was performed using SPSS software (Version 20.0; Chicago, Illinois). Univariate analysis was performed using frequencies for descriptive statistics. Chi-square and Fisher’s exact test were used in the analysis of categorical variables. Wilcoxon Signed-Rank Test was used in the analysis of paired variables. Correlations were considered significant if *p* values were less than 0.05 (two-sided).

## Results

A total of 11 patients (10 female patient and 1 male patient) with high-grade lumbosacral spondylolisthesis met the including criteria were included in our study. All of the high-grade spondylolisthesis in our series occurred at the L5-S1 level. The demographic data is demonstrated in Table 1. The median follow-up time was 36 (26–45) months. There were one case of grade 4 and 10 of grade 3 spondylolisthesis. The average age of patients was 37 (16–69) years, and the median body mass index was 22.95 (18.3–29.7) kg/m^2^. The median blood loss was 300 (100–900) mL, median surgical time of the two-stage surgery was 386 (165–555) minutes, and the median hospital stay was 7 (6–9) days.

**Table 1:** Demographic of 11 patients with high-grade spondylolisthesis

Variables	Number (% or range)
Gender	
M	1 (9%)
F	10 (91%)
Grade	
III	10 (91%)
IV	1 (9%)
Age	37 (16–69) years
Body mass index	22.95 (18.3–29.7) kg/m^2^
Follow-up time	36 (26–45) months
Blood loss	300 (100–900) mL
Operation time	386 (165–555) minutes
Hospital stay	6 (6–7) days
Fusion cage	
TM	5 (45.5%)
Syncage	6 (54.5%)

*TM* Zimmer Trabecular Metal TM-400 (Zimmer Biomet, Warsaw, US) or SynCage (DePuy Synthes, Synthes GmbH, Switzerland).

The radiographic outcomes of 11 patients are shown in Table 2. Compared to pre-operative data, significant improvements were observed in slip percentage, Dubousset’s LSA, LL, and PI-LL mismatch (*P* < 0.05) in postoperative data. Improvements were also observed in pelvic tilt, pelvic incidence, sacral slope, and SVA. However, these changes did not reach statistical significance.

**Table 2:** Changes in pre- and postoperative radiographic parameters pre-operative and postoperative data

	Pre-operative (*n* = 11)		Postoperative (*n* = 11)		*p* value
Local parameters					
Slip percentage	60.0	(53.0, 62.0)	30.0	(23.0, 36.0)	0.003**
Dubousset’s lumbosacral angle	84.1	(75.6, 92.3)	97.9	(92.5, 111.4)	0.003**
Pelvic parameters					
Pelvic tilt	24.2	(22.2, 30.5)	25.4	(15.8, 30.4)	0.131
Pelvic incidence	62.1	(53.1, 80.8)	64.9	(52.1, 81.4)	0.059
Sacral slope	32.3	(30.4, 49.1)	41.9	(35.9, 52.2)	0.091
Spinal parameters					
LL	−49.4	(−70.3, −34.7)	−57.7	(−70.0, −47.1)	0.013*
PI-LL mismatch	16.0	(10.0, 26.3)	9.9	(0.0, 19.0)	0.021*
SVA (mm)	36.9	(18.3, 59.8)	23.6	(0.1, 54.1)	0.213
Wilcoxon signed rank, median (IQR). *P < 0.05, **P < 0.01					

Abbreviations: *LL* Lumbar Lordosis; *PI* Pelvic Incidence.; *SVA* sagittal vertical axis

The functional outcomes of 11 patients are shown in Table 3. Significant improvements in radiographic outcomes were observed in all functional scores including EQ-5D, VAS, and ODI (*P* < 0.05).

**Table 3:** Changes in pre- and postoperative radiographic parameters

	Pre-operative (*n* = 11)		Postoperative (*n* = 11)		*p* value
EQ5D					
	12.0	(10.3, 12.0)	7.0	(5.0, 9.75)	0.018*
VAS					
	9.5	(8.0, 10.0)	2.5	(0.0, 6.0)	0.018*
ODI					
	59.4	(54.4, 65.6)	22.22	(7.2, 49.2)	0.017*
Wilcoxon signed rank, median (IQR). *P < 0.05, **P < 0.01					

*EQ-5D* European Quality of Life questionnaire *ODI* Oswestry Disability Index,*VAS* visual analogue scale.

**Fig. 4:**
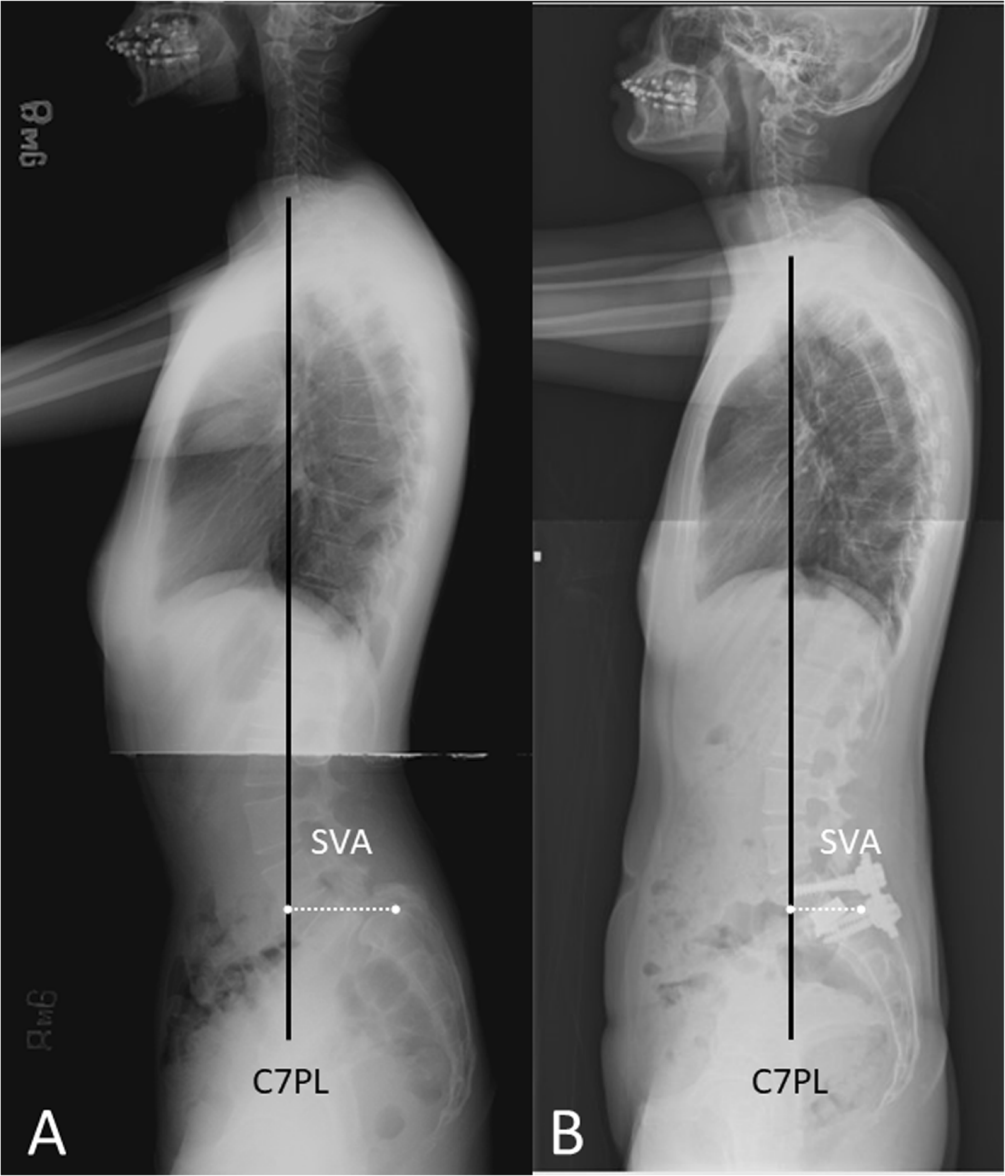
Pre-operative and postoperative standing lateral full-length radiographs of a representative case. The C7 plumb line (C7PL, black line) and the sagittal vertical axis (SVA, white dotted line). **A** Pre-operative standing lateral full-length radiography. **B** After surgery, the C7PL was moved backward through the sacrum with a decreased SC7D

The radiographic changes on the lateral view of spine are displayed in Fig. 4. The SVA had improved from 60.7 mm before surgery to 42.6 mm at the final follow-up; excellent spine sagittal balance was achieved in this patient. Throughout the surgery, no iatrogenic tears of the dura, vessel injury, or peritoneum tears occurred.

One patient developed neurological deficits after surgery; computed tomography indicated right lateral stenosis caused by a fragment of the pars interarticularis. Symptoms were alleviated after additional posterior hemilaminectomy decompression. All 11 patients achieved inter-body fusion in the reduced position within 9 months postoperatively. No screw loosening, cage dislodgement, or other type of implant failure had occurred.

According to the modified MacNab criteria, 6 patients (55%) achieved an excellent result, 4 patients (36%) a good result, and 1 patient (9%) a poor result. Ten patients (91%) were satisfied with this procedure (Table 4).

**Table 4:** Clinical outcomes according to modified Macnab criteria

Result	Patients (n)	Rate (%)	Criteria
Excellent	6	55	No pain; no restriction of mobility; return to normal work and level of activity
Good	4	36	Occasional nonradicular pain; relief of presenting symptoms; return to modified work
Fair	0	0	Some improved functional capacity; still handicapped and unemployed
Poor	1	9	Continued objective symptoms of root involvement; additional operative intervention needed at the index level irrespective of length of postoperative follow-up

## Discussion

The aim of this study was to investigate the surgical outcomes of the anterior cantilever procedure followed by ALIF and posterior mono-segment instrumented fixation for the treatment of high-grade spondylolisthesis. Our findings suggest that this procedure is a valid technique for improving slip percentage, lumbar lordosis, Dubousset’s LSA, PI-LL mismatch, and functional status for high-grade spondylolisthesis patients with a low complication rate and blood loss.

Traditionally, surgical reduction of high-grade spondylolisthesis can only be achieved via the posterior approach [9, 16–23]. Min et al. described single-stage posterior reduction with sacral dome resection in 15 patients, reporting that slip percentage had improved from 94 to 23% [16]. Shufflebarger et al. reported a slip percentage improvement from 77 to 13% in 18 patients [18]. Although greater percentages of slip reduction compared with that achieved in our study have been reported, large wounds, extensive back muscle detachment, massive osteotomies, and excessive neural retraction are inevitable, which may increase postoperative pain and recovery time [24, 25]. Complications rates of pseudarthrosis and neurologic injury as high as 13 and 48%, respectively, have been reported [26].

Poor clinical results and high complication rates have been reported, especially in patients with extremely narrow intervertebral spaces [27–31]. In these patients, the severe adhesion caused by scar tissue is hard to remove and it is difficult to distract the vertebral bodies by the pedicle screw system alone [32–35]. The excessive distraction and sharing force from high LSA may cause loosening of the pedicle screw and vertebral fractures [36]. Thus, longer instruments for fixation of L4–5-S1 are usually necessary to hold the reduction position in high-grade spondylolisthesis when using posterior approach procedures [22]. However, in our cases, a high fusion rate and a low implant failure rate were achieved using mono-segment instrumented fixation alone. The reason may be that the anteriorly-inserted large lordotic cage shifts the shearing force to a compression force between L5-S1, and provides a relatively larger contact area to improve the inter-body stability [37, 38].

The anterior approach for surgical treatment of spondylolisthesis was first mentioned by Capener in 1932 [39]. In 1979, Bradford performed 10 cases of combined posterior and anterior reduction of spondylolisthesis by means of an anteriorly placed plate, which engages two screws. In Bradford’s surgery, posterior decompression was performed first to remove the obstruction to reduction, then, anterior reduction and plating were performed. However, performing decompression first may increase the risk of nerve injury. Employing this technique, root injuries in up to 30% of cases were recorded [40].

**Fig. 5:**
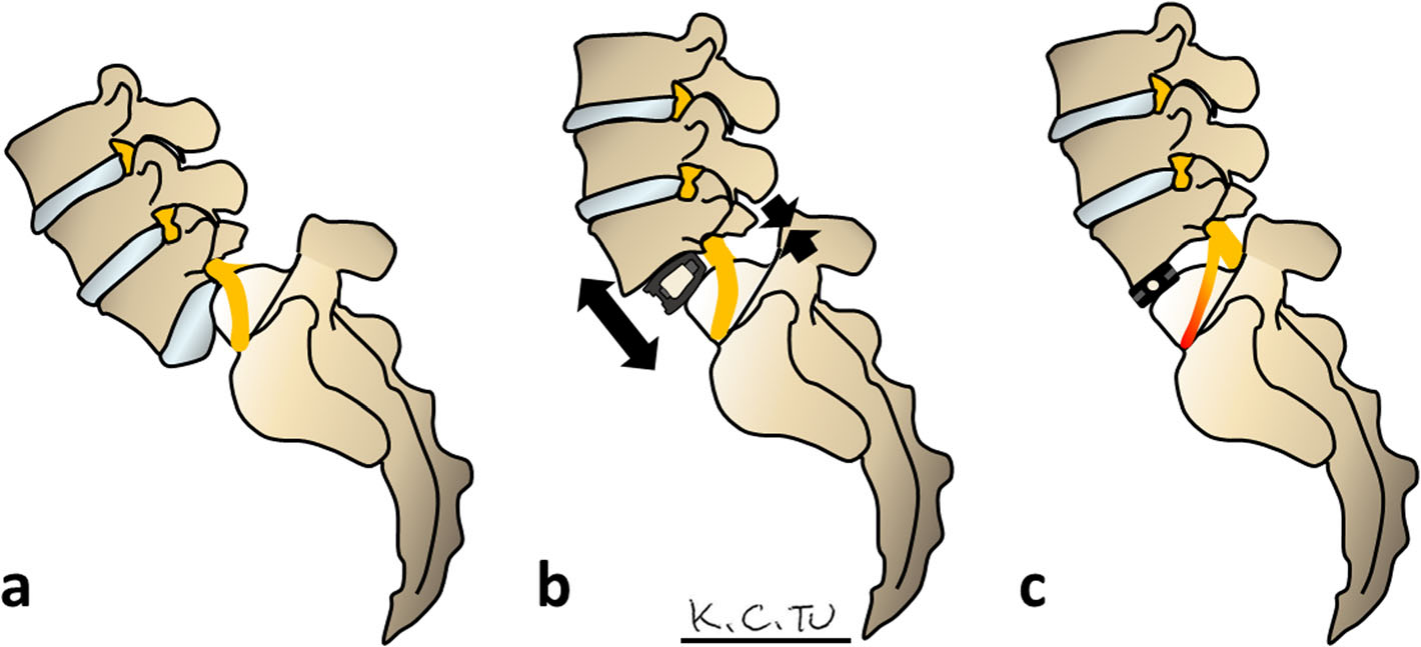
The picture demonstrates the anterior approach with partial slip reduction and the posterior approach with near complete slip reduction. **A** L5-S1 high-grade spondylolithesis (**B**) ALIF lordosis cage provides sufficient anterior lengthening and posterior shortening (black arrow), achieving partial reduction, increasing LSA, and reducing L5 nerve root traction injury. **C** The posterior approach can almost completely reduce slips, but insufficient LSA and lordosis may cause L5 nerve root traction injury. Posterior instrumentation has been omitted from the above pictures

To solve this problem, we developed the anterior cantilever procedure for surgical reduction. In our technique, the anterior approach with cantilever technique was performed before decompression (Fig. 1B and C). A trial cage was used to dilate the disc space and mobile posterior element (Fig. 1G). Consequently, a partial reduction was easily and safely achieved using a Cobb elevator. Additionally, the anteriorly-inserted lordotic cages provided adequate anterior lengthening and posterior shortening, resulting in increased LSA and decompression of the nerve, thereby decreasing the risk of L5 root stretch injury, which may occur in posterior reduction procedures (Fig. 5).

To the best of our knowledge, this is the first study to describe the detailed surgical technique and the surgical outcomes of anterior cantilever technique and ALIF for the treatment of high-grade spondylolisthesis. With minimally invasive nature, ALIF and percutaneous screw fixation has become increasingly popular for the treatment for spondylolisthesis in the present time. Lee et al. performed ALIF and posterior percutaneous pedicle screw fixation in 73 patients. They reported a satisfactory outcome rate of 94.5%, a fusion rate of 97.3%, and a complication rate of 8.2% [11]. Kim and Lee performed ALIF alone in 20 patients and reported a satisfactory outcome rate of 85% and a fusion rate of 90% [41]. Our satisfactory outcome rate (91%), fusion rate (100%), and complication rate (9%) were similar to those of studies in which low-grade spondylolisthesis was treated with ALIF alone or ALIF with posterior instrumentation. In our study, the fusion rate (100%) is relative high among the similar studies [10–13, 41]. The reason may be that all of our cases undergone posterior instrumentation. The intervertebral body fusion is not the only way to obtain absence of motion, it can be guaranteed also by pedicular screws and bars. This suggests that followed by ALIF and posterior mono-segment instrumented fixation is an effective and safe procedure for treatment of high-grade spondylolisthesis.

There are two main challenges when performing this technique. First, the common iliac vessels may be injured during the ALIF procedure. There should be an experienced vascular surgeon standing by during the procedure. Second, there may be a risk of cage dislodgement while repositioning the patient into the prone position. Two 3.5-mm cancellous screws at the anterior superior corner to serve as prophylactic anti-dislocation screws are sometimes needed in our experience. (Fig. 3C).

Although complete reduction was not achieved in our series, correct sagittal alignment and restoration of the LSA, which are more important for decreasing the shear force and increasing the fusion rate, could be achieved [42]. In a cadaveric study by Petraco et al. [43], while L5 reached full reduction, the strain per increment of reduction increased rapidly, subsequently increasing the risk of stretch injury to the L5 nerve; partial reduction may thus be safer than complete reduction. In our cases, the average postoperative LSA was 97.9°, which successfully corrected the lumbosacral kyphosis to lordosis and was in the range of postoperative LSA, which was treated with posterior reduction procedures (96–106°) [16, 22, 44].

In a literature review of surgical reduction of high-grade spondylolisthesis, posterior reduction resulted in higher blood loss than in situ fusion (584 mL vs 451 mL) [26]. In our study, blood loss mainly occurred during the procedure for posterior mono-segment instrument fixation. The average blood loss was 300 mL in our study, which was less than that of in situ fusion via posterior approach.

This study has several limitations, as well as bias, which needs to be corrected in future studies. First, due to the retrospective nature of this case series, we did not have a control group and some parameters that may affect the clinical results, such as mental status and bone mineral density, were not included in our analysis. Second, the case number was small in our study, and thus some parameters, such as pelvic tilt, pelvic incidence, sacral slope, and SVA did not reach statistical significance, future studies with larger case numbers are warranted to investigate the surgical effects of these parameters. Third, neurophysiologic monitoring was not used during the anterior cantilever procedure in our study because neurophysiologic monitoring equipment was not available 10 or more years ago. Nevertheless, ALIF was reported to have a low possibility of neural injury during the operation [11]. Only one patient developed neurological deficits after the surgery. Neurophysiologic monitoring equipment should be used during high-grade spondylolisthesis procedures in future surgeries.

## Conclusions

Anterior cantilever procedure followed by ALIF and posterior mono-segment instrumented fixation is a valid procedure for treating high-grade spondylolisthesis. This novel technique achieved a high fusion rate, partially reduced slippage, and significantly improved lumbosacral angle, while minimizing common complications, such as pseudarthrosis and nerve traction injury, as well as excessive soft tissue dissection and blood loss in posterior reduction procedures. However, posterior instrumentation is still required to the structural stability of the ALIF procedure.

## Data Availability

The data that support the findings of this study are available from the corresponding author upon reasonable request.

## Abbreviations

LSA: Dubousset’s lumbosacral angle
SS: Sacral slope
PI: Pelvic incidence
PT: Pelvic tilt
LL: Lumbar lordosis
SVA: Sagittal vertical axis
